# Epitome of Skin Diseases, with Formulæ for Students and Practitioners

**Published:** 1883-12

**Authors:** 


**Epitome of Skin Diseases, with Formulæ for Students and Practitioners.** By the late TILBURY FOX, M. D., F. R. C. P., and T. COLCOTT FOX, M. B., M. R. C. P. Third American edition by T. COLCOTT FOX, B. A., M. B., Physician for Diseases of the Skin to the Westminster Hospital, etc. Philadelphia: H. C. Lea's Son & Co. 1883.

As a manual for the student and as a book for ready reference to the practitioner this work has long been highly esteemed. The present edition has been completely revised and will be increasingly useful and acceptable. The classification of dermal diseases, adopted by the American Dermatological Association has been introduced. For students particularly who have not time to read the larger treatises of Duhring and other American authors, it is a most desirable work.

